# In Vitro Investigation of the Fixation Performance of a Bioabsorbable Magnesium ACL Interference Screw Compared to a Conventional Interference Screw

**DOI:** 10.3390/life13020484

**Published:** 2023-02-10

**Authors:** Nad Siroros, Ricarda Merfort, Yu Liu, Maximilian Praster, Frank Hildebrand, Roman Michalik, Jörg Eschweiler

**Affiliations:** Department of Orthopaedics, Trauma, and Reconstructive Surgery, University Hospital RWTH Aachen, 52074 Aachen, Germany

**Keywords:** ACL reconstruction, interference screw, bioabsorbable, magnesium, biomechanics

## Abstract

An anterior cruciate ligament (ACL) reconstruction is a common treatment for patients with ACL rupture that aims to regain pre-injury knee stability and kinematics. During the ACL reconstruction, one method to fix the graft is the use of an interference screw (IS). The IS should provide initial stability and secure the graft during the healing period. In recent years, magnesium has emerged as an alternative material to permanent metal and polymer ISs. In addition, differences in designs, such as the shape of the IS, can influence the fixation performance of the IS. Therefore, in this biomechanical experiment, two different screw designs with two ligament materials were compared in an insertion and a pull-out test at a rate of 1 mm/s. The screw designs were a conventional polymer screw and a magnesium screw. Porcine tendon and nylon rope were used as ligament materials. All tests were performed in polyurethane foam blocks with 15 PCF density (Synbone AG, Switzerland). As a result, both screw designs required an insertion torque of less than 3 Nm. There was a significant difference between the porcine and nylon rope in pull-out tests for each screw design. The magnesium screw had the highest pull-out force at 412.14 ± 50.00 N for porcine tendon and 707.38 ± 21.81 N for nylon rope. There were no significant differences in tunnel widening (narrow–wide ratio) between each ligament material. The magnesium screw showed the lowest narrow–wide tunnel ratio, implying a better ability to compress the graft to the tunnel. In conclusion, a more optimized magnesium IS design resulted in better graft fixation and an improved ACL reconstruction outcome.

## 1. Introduction

The anterior cruciate ligament (ACL) is one of the most important ligaments in the knee, influencing stability and kinematics. It is the most commonly injured ligament in the knee [1]. The ACL originates from the medial of the lateral femoral condyle and inserts into the tibia plateau medial to the anterior horn of the lateral meniscus [2,3]. It is a single continuum of fibers which is sometimes separated into anteromedial and posterolateral bundles depending on the attachments of the fibers to the tibia [2]. In the case of ACL rupture, ACL reconstruction aims to regain the knee’s stability. Although the result is between 75% and 97% satisfactory, failures may occur due to traumatic, technical, and biological reasons [4,5,6].

One factor that affects the ability to return to sport is the surgical technique [4]. The type of graft fixation is chosen depending on the surgeon. A survey conducted in Germany showed that 81% of surgeons prefer a monofixation in tibia fixation, and 60% of surgeons use bioabsorbable screws [7]. Methods of mechanical fixation can be categorized as either direct or indirect. In the case of indirect fixation, such as with a cross-pin, fixation and endobuttons suspend the graft within the bone tunnel [2]. For direct fixation, interference screws (ISs), staples, and spiked washers compress the graft directly against the outer surface of the wall of the bone tunnel [2]. The IS fixes a soft-tissue graft within a bone tunnel. IS screws can be made of either non-degradable or bioabsorbable material. A permanent IS can be a metallic (i.e., stainless steel and titanium) or a non-biodegradable polymer such as PEEK [8].

A meta-analysis comparing bioabsorbable and metallic IS fixation in ACL reconstruction in ten studies with a total of 790 patients found no statistical differences in infection rate, arthrometer testing, pivot-shift testing, International Knee Documentation Committee final score, and Lysholm score [9]. Moreover, a comparison of magnesium and polymer ISs with a similar screw design showed no statistical difference in anterior tibial translation [5]. Apart from the material of the IS, a comparison of three bioabsorbable magnesium IS designs showed that screw designs 1, 2, and 3 had different pitches of 2.5 mm, 2.25 mm, and 1.5 mm, respectively, and pitch depths of 0.8 mm, 0.8 mm, and 0.5 mm, respectively. The results found differences in fixation performance, but they were not statistically significant [10]. However, Athwal et al. showed that screw tip design has an influence on the fixation performance [11], and the study of the IS body slope showed that it also influenced the fixation strength [12]. The use of a permanent metal IS has some disadvantages, including graft damage, tunnel widening, imaging modalities, required implant removal surgery, and increased difficulty in revision surgeries [5]. However, magnesium has proven to be a good alternative due to its biocompatibility, bioabsorbability, and strength [5,9,13]. The shape of the screw, including diameter, length, and thread can influence the fixation strength. An appropriate screw diameter and length to the tibia tunnel results in optimum fixation strength [14] and less graft slippage [15]. Hence, interference screw design and surface conditions can influence the ACL fixation performance.

Since the biomechanical testing of fixation by interference screws has shown fixation failures [2,16,17], the ACL IS testing criterion is the the fixation performance. The IS must provide sufficient initial graft fixation while being biocompatible with the human body. In addition, the optimum fixation condition involves some parameters such as suitable tunnel diameter and screw diameter and length, as well as graft size and material. This fixation condition directly influences the insertion torque [18]. It is also recommended that the insertion torque be included in the IS fixation test [2] and range between approximately 0.5 and 3.5 Nm [19]. 

An initial evaluation of the screw insertion and fixation performance can provide an impression of the screw implementation and is, therefore, a good indication of a well-designed IS. The testing method for ISs is adapted from the guideline for the in vitro testing of the cruciate ligaments and ligament reconstruction [20] and the standard specification and test methods for metallic medical bone screws (ASTM F543). The initial fixation strength is determined by a pull-out test, in which the pull-out rate ranges between 5 and 1000 mm/min [21]. For the biomechanical test, the tunnel size is suggested to be equal to or 1 mm larger than the diameter of the screw [14,15,22]. The testing bone material for the biomechanical test can be varied, for example, polyurethane block, bone biomechanical model, or cadaver [5,11,23,24,25,26]. The polyurethane foam block for biomechanical testing should match the material standard (ASTM F1839). The foam block is suitable for an initial evaluation of the screw due to its cost and ease of use compared to the biomechanical bone model and cadaver. 

In addition to the pull-out test as initial evaluation, observation of the tunnel widening (the change in shape of the tunnel) can be performed. Since the size of the IS combined with the graft is normally larger than the diameter of the tunnel, the compression occurring in the fixation can cause the tunnel to widen. The observation of the tunnel widening should imply how the screw functions towards the fixation (progression to the tunnel wall and the interaction with the graft).

**Figure 1:**
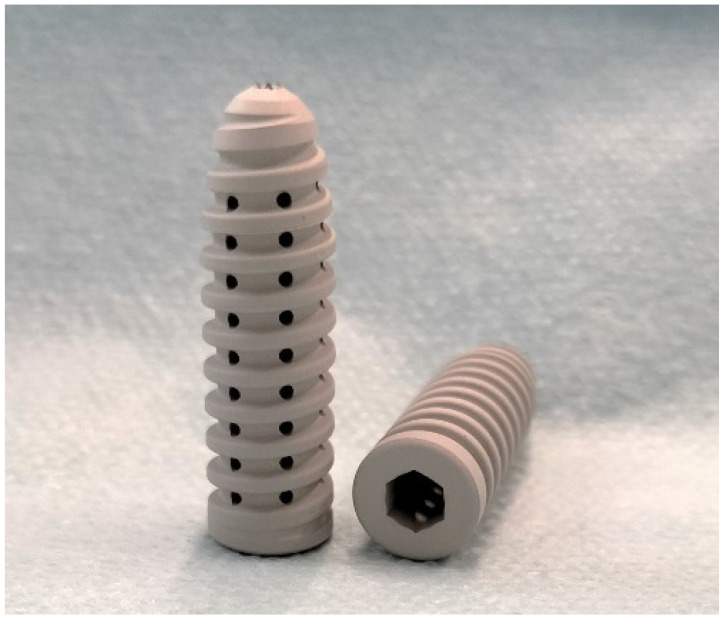
Illustration of a bioabsorbable magnesium interference screw.

Therefore, in this study, a magnesium IS design, see Figure 1, is used for comparison with a conventional polymer IS design. The objective of this study is the testing and comparison of the insertion and fixation performance of the two different IS designs, focusing on the evaluation of the material surface and the shape of the screw. The tests also assess how closely a construct’s biomechanical performance matches that of the normal fixation immediately after surgery [2]. 

## 2. Materials and Methods

The experiment consisted of two parts, the insertion and the pull-out test, which were carried out one after the other for all samples. A bioasorbable magnesium IS (n = 10) was used for comparison with a conventional polymer IS (n = 10). A total of three 15 PCF polyurethane foam blocks (Synbone AG, Switzerland) were selected to represent the bone material. Porcine flexor tendon and nylon rope were selected to represent the ACL graft for the experiment. Porcine tendon is a common material for representing ACL material. Furthermore, since this was an initial evaluation of the ISs, nylon rope was used as an additional material, aiming to eliminate any possible inconsistency in porcine tendons, such as those due to graft size, ligament stretch, deformation, or damage during the test, that could directly effect the full performance (mainly focusing on the fixation performance) of the screw. The rope was considered rigid and was not subjected to large strains. Therefore, this nylon rope set-up did not represent the actual implementation condition of the screws but determined the performance of the screw with the least influence on the non-focused parameter.

The test set-ups were adapted, as mentioned previously, from the in vitro ligament testing [20] and the bone screw testing standard (ASTM F543). During each test, the insertion torque was confirmed if it satisfied the insertion torque limit. In the pull-out test, the maximum pull-out force and tunnel widening were recorded and analyzed.

### 2.1. Material and Specimen Preparation

The experiment consisted of two IS designs (polymer IS and magnesium IS) tested with a porcine tendon and a nylon rope as an ACL replacement alternative, creating four groups for the test set-up. Within each group, the tests were performed five times. Table 1 shows an overview of the test group and the number of tests in each group.

**Figure 2:**
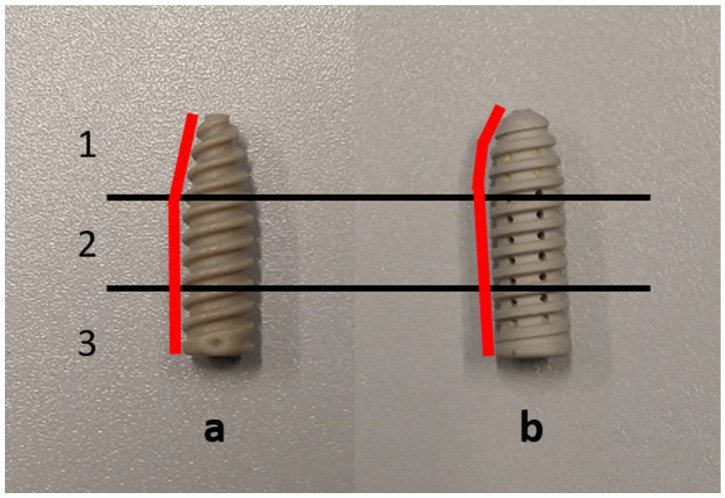
Illustration of the slope profile different of (**a**) polymer IS and (**b**) magnesium IS with red lines indicating the body slope of each screw and the block line dividing the screw into three regions (1, 2, and 3).

All ISs had a diameter of 9 mm and were 30 mm in length. The pitch and pitch depth of each screw design were measured using a Vernier caliper (ACCUD Co., Ltd., SuZhou, China). The pitch was measured at 2.5 mm in both screw designs. The pitching depth for the polymer and magnesium screws was 1 mm and 0.825 mm, respectively. In Figure 2, for visual observation, the screws are separated into three sections (1–3). The red line shows the body slope profile from the tip (1) to the head (3) of both IS designs.

A porcine flexor tendon and a 6 × 9 mm width elliptical-shaped nylon rope were selected as the ACL material for the test. Porcine feet were ordered from a local butcher. Therefore, no ethical approval was required. The porcine flexor tendon was harvested and stored in phosphate-buffered saline (PBS) and frozen at −16 °C. The ligaments were thawed to room temperature before the experiment. A total of ten porcine tendons with a size averaging 7.86 ± 0.63 mm in width and ten nylon ropes were prepared.

The 15 PCF solid rigid polyurethane foam blocks were cut into seven smaller 11 × 3.5 × 4 cm^3^ blocks. In each block, three holes were drilled. Tunnels of 9 mm and 10 mm diameter were drilled for the porcine tendon and nylon rope test, respectively, as these tunnel sizes proved suitable in the initial tests. Each tunnel diameter was measured and recorded.

Before the experiment, the ligament clamp was connected to the testing machine with a 2.5 kN load cell (DYNA-MESS Prüfsysteme GmbH, Stolberg, Germany). Additionally, at the beginning of each test, a ligament specimen (either porcine tendon or nylon rope) was fixed to a ligament clamp for the insertion and pull-out test.

### 2.2. Insertion Test

**Figure 3:**
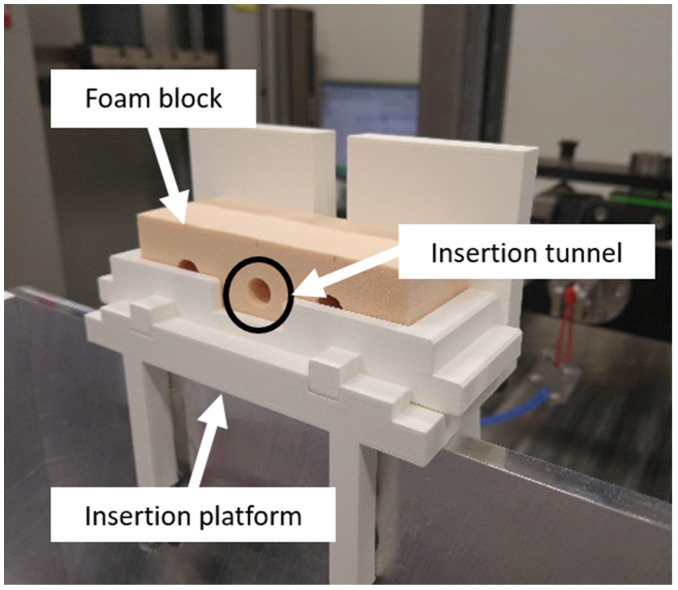
The insertion test set-up where the foam block was fixed to the 3D-printed insertion platform, preventing the block from moving. The black circle shows the open side of the insertion tunnel.

The screw insertion was performed by fixing the bone block to a 3D-printed screw insertion platform, preventing the bone block from moving (Figure 3). 

**Figure 4:**
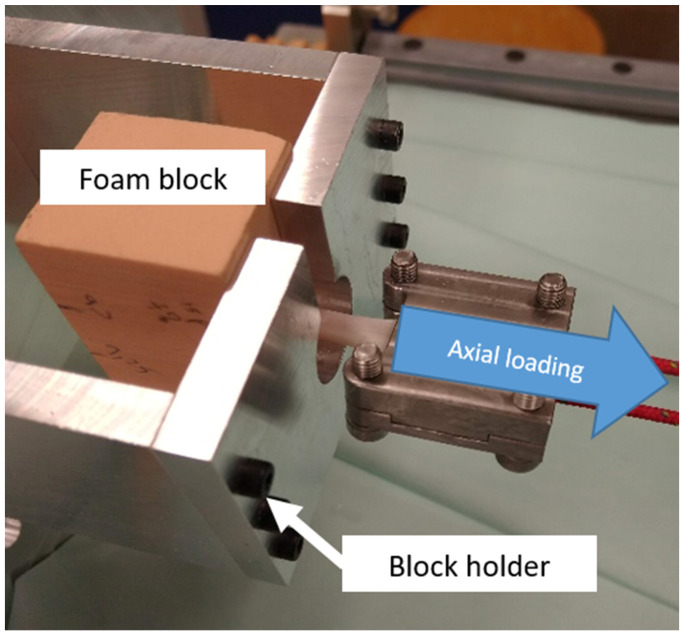
The pull-out test set-up. The foam block was placed in the block holder. The ligament material was connected to the machine using a ligament clamp and axially pulled away from the block.

After the ligament material was fixed to the ligament clamp, the free end was pulled through the bone block tunnel (insertion tunnel) from the back to the open side of the tunnel. The ligament was pulled, and the screw was inserted to fix the ligament to the tunnel. The insertion torque was measured using a digital torque screwdriver (TSD-400, Checkline by ELECTROMATIC Equipment Co., Inc., Lynbrook, NY, USA) to verify that the insertion torque met the target of less than 3 Nm insertion torque. After the insertion was performed, the foam block with implemented IS was removed and then placed in a block holder (Figure 4).

### 2.3. Pull-Out Test

The pull-out test was performed by placing the implemented IS in a block holder with the tunnel axially aligned to the pulling direction, as shown in Figure 4.

The pull-out test was performed at a rate of 1 mm/s until the machine reached a set travel limit of 96 mm. The first peak force was recorded as the maximum pull-out force. The modes of failure, such as ligament material slippage, screw loosening, and screw breakage, were observed. The determination of the mode of failure was as follows:

- Ligament material slippage: when the ligament slips along the tunnel where the screw remains intact in the tunnel;
- Screw loosening: when the screw loses its interaction with the tunnel wall and moves out or within the tunnel;
- Screw breakage: when the material of the screw cannot withstand the load and breaks/fails.

**Figure 5:**
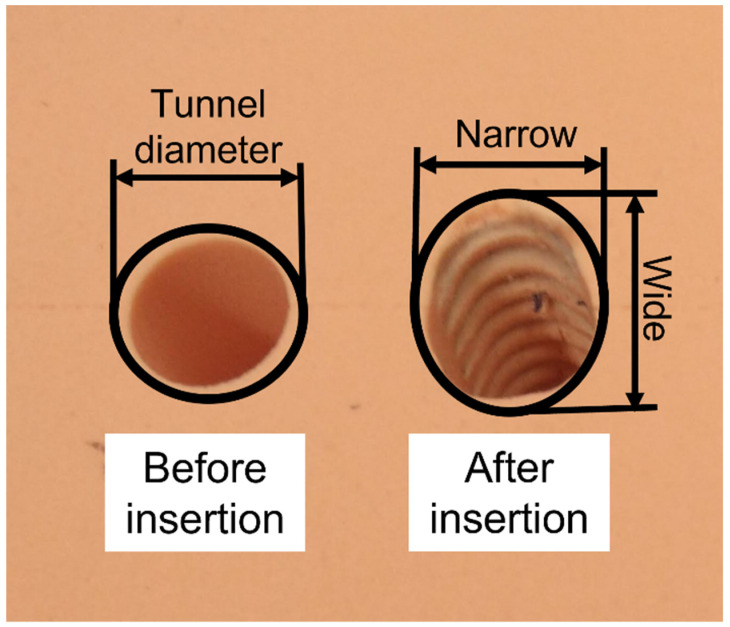
Tunnel shape at the insertion site of the bone block before (**left**) and after (**right**) the insertion.

After the pull-out test, the screw and the ligament were removed from the tunnel. The tunnel was measured at the insertion site. Since the tunnel was widened to an eliptical shape, the measurement consisted of the narrow (minor axis) and wide (major axis) sides of the tunnel. Figure 5 shows the determination of the tunnel diameter before the insertion and the narrow–wide side after the insertion. To compare the effect of the two IS designs with two ligament materials and two tunnel sizes, the narrow–wide ratio of the the tunnel was introduced. The ratio represented the tunnel widening pattern of each test set-up. This showed the interaction between the tunnel, graft, and screw. A lower ratio, for example, 0.9, means that the tunnel is more elliptical than when the ratio is closer to 1.

### 2.4. Statistics

Statistical analysis was conducted for the mean and standard deviation. Each test set-up was analyzed using a two-tailed, two-sample *t*-test. The variance of each set-up was checked to determine an equal or unequal variance *t*-test. Statistical significance was determined at *p*-values < 0.05.

## 3. Results

The insertion torque of both screw designs and ligament materials satisfied the goal of not exceeding 3 Nm insertion torque. The modes of failure were all caused by ligament material slippage in all experiments. Table 2 shows the comparison of the pull-out force of each test group (n = 5). From the results shown, it can be seen that the nylon rope had significantly higher pull-out forces in all screws compared to the porcine tendon (*p* < 0.05). Moreover, for both ACL materials, the pull-out force of the magnesium screw design was significantly higher than that of the polymer screw design (*p* < 0.05).

In addition to the pull-out force from the experiment, the tunnel widening pattern, described as a narrow–wide ratio, was observed. Figure 5 illustrates an example of the tunnel shape at the insertion site before and after the insertion. The tunnel diameter was measured before insertion. After the test, the tunnel widening pattern could be observed. The compression of the screw and the ACL material caused the tunnel to widen, resulting in an elliptical tunnel shape at the insertion site. These were described as the “narrow” and “wide” sides of the tunnel. The ratio between the narrow and wide sides is called the “narrow–wide” ratio.

Table 3 shows the tunnel measurement and tunnel widening pattern for each version of the interference screw. Firstly, in both screw designs, the sample size of four (n = 4) in the test using porcine tendon occurred due to measurement record error; therefore, only four measurement data were evaluated. Secondly, the magnesium screw showed no significant difference in the tunnel widening pattern (narrow–wide ratio) between using porcine tendon and nylon rope (*p* = 0.49), while the polymer screw showed a significant difference between the two ligament materials (*p* < 0.05). When comparing the tunnel widening pattern between the screw designs, there was a significant difference in the porcine tendon test (*p* < 0.05), and in the nylon rope test (*p* = 0.05). The tunnel widening ratio allowed the results of the two different tunnel diameter to be compared. The narrow–wide ratio of each test set-up is discussed in the next section.

## 4. Discussion

Biodegradable screws have been developed as an alternative to metal [2]. The advantages include the absence of signal artifacts during imaging and an easier revision procedure [2,27]. To achieve the objective of comparing the insertion and fixation performance of two different IS designs, a test set-up and procedure were adapted from the guideline for the in vitro testing of the cruciate ligaments and ligament reconstruction [21] and partially from the standard specification and test methods for metallic medical bone screws (ASTM F543). A test set-up using foam blocks, porcine tendons, and the IS was performed to set the benchmark values.

Before the test, all screw designs were visually investigated. The diameter, length, and pitch of both screws were the same except for a shallower pitch depth of the magnesium screw compared to the polymer screw. One finding was that the finishing surface of the polymer screw was smoother than the surface finish of the screw made of magnesium due to the material surface treatment.

The first assessment of this study was the assessment of the insertion torque. The insertion torque limit was set at 3 Nm based on the literature, which states that (1) insertion torque ranges between 0.5 and 3.5 Nm [19], (2) in insertion torque studies of various IS types, when excluding titanium ISs, the insertion torque can be within a 2.5 Nm range [18,28], (3) insertion torque exceeds 2.5 Nm, which is related to having an optimum fixation condition [18]. 

The evaluation of the IS performance focused on the investigation of the surface condition and the shape of the screw. In the pull-out test, the magnesium IS showed significantly higher pull-out force than the polymer IS in both porcine tendon and nylon rope cases. This means that, in the same fixation condition (same tunnel size and ACL material), the magnesium IS provides better initial fixation strength compared to the polymer IS. However, this conclusion cannot be applied when comparing the porcine tendon and nylon rope of the same IS because of the difference in the fixation condition (different tunnel size and ACL material). One observation is that, for the polymer IS, the pull-out force increased by 216 N when using nylon rope, while, for the magnesium IS, it increased by 238 N. The 20 N difference could imply an effect of the material surface condition since the nylon rope set-up created more friction with the IS. Therefore, the surface condition reflects more in this set-up than the more slippery porcine tendon set-up. For this case, it could be implied that the shape of the IS influences the fixation strength more than the surface condition.

Regarding the evaluation of the shape of the IS, it was found that, in the literature, there was no significant difference in pull-out behavior for ISs with the same material with different pitches and pitch depths of 1 mm and 0.3 mm, respectively [10]. The pitch and pitch depth parameters may not be a major factor influencing the fixation strengths since magnesium only had a pitch depth difference of 0.175 mm compared to the polymer screw but had significantly higher fixation strength. Moreover, the use of the same screw design with a modification of the tip significantly affected the fixation performance [11], implying the importance of the tip portion of the screw as well as the importance of its body slope. The shape of the polymer IS had a higher slope profile from the head to the tip of the screw than the magnesium screw; however, at the screw head, they shared the same diameter of 9 mm (Figure 2). Since the length of all screws was the same, the effect of the screw length on the fixation strength [14] can be neglected, and the effect of the body slope can be focused on. 

Daneshvarhashjin et al. [12] investigated the effect of the body slope profile of the interference screw on the initial stability of the ACL reconstruction and suggested (1) avoiding high contact pressure from the tip to one-third of its length, and (2) a proper graft fitting is essential in the last two-thirds of the screw length since major graft slippage occurs in this region. Moreover, a tunnel diameter equal to or 1 mm larger than the screw diameter provides a better fixation compared to a 1 mm smaller tunnel diameter [22]. Therefore, the magnesium screw satisfied the two suggested points of Daneshvarhashjin et al. [12]. This implies a higher pull-out force in the magnesium screw than in the polymer screw. The tunnel widened in the magnesium screw, showing its ability to compress the ligament eccentrically to the bone polymer screw due to its lower narrow–wide ratio of 0.92 compared to 0.94 for the other two screw designs. The results also implied that, in a multiple-strands graft, the magnesium screw also compresses the grafts directly to the tunnel surface more, thus, fixing them better.

The foam block used for the testing complied to the ASTM F1839 standard. A 9 mm tunnel and screw diameter was an optimum set-up for the IS implementation since the graft slippage is the lowest when the screw and tunnel have the same diameter [15]. However, when using nylon rope to achieve similar ligament interaction, a 10 mm diameter tunnel was selected. A tunnel diameter of 9 mm and 10 mm is congruent to the literature that states that the tunnel diameter should be equal to or 1 mm larger than the IS diameter [14,15,22]. The test set-up where the pull-out force was aligned axially to the tunnel created the “worst-case scenario”; however, this is not related to any biomechanical motion of the knee [23]. 

One additional observation is the tunnel widening pattern after the insertion. The insertion results of the magnesium IS show that the nylon rope had a smaller deviation, and there was no significant difference in tunnel widening compared to porcine tendons. The tunnel widening pattern showed the interaction of the bone–screw–ligament. The ratio of approximately 0.92 in the magnesium IS shows that the IS compressed the ligament directly to the tunnel wall, resulting in an elliptical tunnel. There was no significant difference in the tunnel widening ratio between the porcine tendon and nylon rope in the magnesium IS cases. This means that, for magnesium ISs, nylon rope can give the same material response as porcine tendon. For the polymer IS, the tunnel widening ratio was higher than that in the magnesium IS cases, which means the tunnel after the insertion was more rounded. In this case, more investigation into the optimum ligament compression influencing the fixational strength should have been conducted.

Despite all efforts, drawbacks still exist in this study. The experiment aimed to compare different IS designs. Since many fixation methods exist, the experimental set-up represented a monofixation with a single band graft. The result is not to be applied to all graft and fixation methods (i.e., hamstring graft, bone–patella tendon–bone grafts). Due to the current set-up, the foam block had to be moved to the block holder. This required more steps during the test. Fixing the bone block in one position may help to smoothen the experimental procedure and reduce the risk of possible errors. As modes of failures, only ligament material slippage, screw loosening, and screw breakage were observed. Additional observation of the nature of graft failure, including transverse rupture or constriction of the graft (and the position of the rupture), could possibly give a more detailed view of the modes of failure. Tunnel widening was only observed at the entry point of the tunnel, not for the overall length of the tunnel. Thus, the tunnel widening did not correctly imitate any tunnel widening after ACL reconstruction or during the rehabilitation process. An additional limitation is that the exact dimension of the screw was not determined but was visually observed. It was shown that parameters such as the body slope had a higher influence on the pull-out force than others such as screw depth and pitch. However, to determine the exact design parameters that influence the fixation performance, further studies have to be conducted.

## 5. Conclusions

Reconstruction of the ACL aims to approximate the mechanical and biological properties of the original ligament. The adequate fixation of the artificial tissue is, therefore, important. It can be concluded that a combination of parameters in screw design, such as the material, surface condition, length, pitch, pitch depth, and slope design, influences the performance of the IS. An optimized design improves fixation and leads to a better ACL reconstruction outcome. Problems such as graft fiber transverse rupture and graft necking can be minimized. Lastly, since the magnesium screw had a promising outcome, it can be further investigated in a different test set-up and protocol such as by a mechanical dynamic test and various implantation approaches. Biodegradable screws should provide sufficient mechanical fixation until adequate biological fixation has been achieved and should then be degraded completely [2].

## Figures and Tables

**Table 1:** Overview of the testing groups and the number of tests.

Screw Design	ACL Replacement Material	
	Porcine Tendon	Nylon Rope
Polymer	5	5
Magnesium	5	5
Total	10	10

**Table 2:** The comparison of the pull-out force of the different screw designs.

		Porcine Tendon	Nylon Rope
**Polymer**	Mean, N	269.14 ± 21.43	469.30 ± 29.35
	*p*-value	<0.05	
**Magnesium**	Mean, N	412.14 ± 50.00	707.38 ± 21.81
	*p*-value	< 0.05	
**Polymer–magnesium** **IS comparison (*p*-value)**		< 0.05	< 0.05

**Table 3:** Tunnel measurement at the insertion site before and after the test and the tunnel widening pattern.

		Porcine Tendon	Nylon Rope
**Polymer**	No. of tests	n = 4	n = 5
	Before (mm)	9.28 ± 0.03	10.06 ± 0.05
	After—narrow (mm)	9.77 ± 0.07	10.13 ± 0.08
	After—wide (mm)	10.02 ± 0.09	10.76 ± 0.11
	Narrow–wide ratio	0.97 ± 0.01	0.94 ± 0.01
	*p*-value	< 0.05	
**Magnesium**	No. of tests	n = 4	n = 5
	Before (mm)	9.08 ± 0.03	10.01 ± 0.07
	After—narrow (mm)	9.48 ± 0.16	10.02 ± 0.07
	After—wide (mm)	10.20 ± 0.35	10.86 ± 0.18
	Narrow–wide ratio	0.93 ± 0.02	0.92 ± 0.01
	*p*-value	0.49	
**Polymer–magnesium** **IS comparison (*p*-value)**		< 0.05	0.05

## Data Availability

Not applicable.
